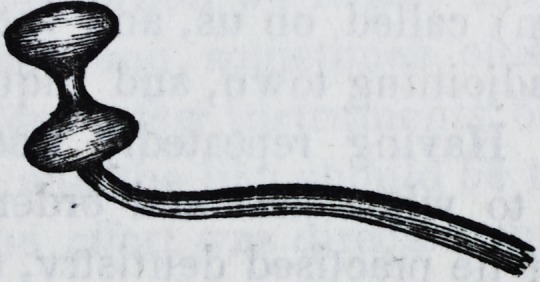# Cleft Palate and Its Treatment

**Published:** 1845-03

**Authors:** S. P. Hullihen

**Affiliations:** Wheeling, Va.


### ARTICLE II.

*Cleft Palate and its Treatment.* By DR. S. P. HULLIHEN, Wheeling, Va.

A CLEFT, or fissure of the palate, is a congenital separation of the parts, with but little if any loss of substance. It is more or less extensive in different cases, always commencing with the velum or soft palate, which is, sometimes, alone involved; but, more frequently it extends to a greater or less degree through the roof of the mouth, and very often through the alveolar arch. The cleft is always at the median line in the soft palate, but, as it continues forwards, it inclines to one side or the other of the septum nasi, or branches off on each side, forming what may be called a double fissure; and when this happens, one or both branches always extends through the alveolar arch. In some cases in which the cleft in the palatine arch is extensive, a considerable turning up of the edges of the divided bones may exist, which contributes greatly to the width of the fissure, the inconvenience of the deformity, and the difficulties of affecting a cure.

The consequences of a cleft palate are complex, and very distressing in their character. It prevents the infant from being nursed at the breast, impedes deglutition in after-life, interferes with mastication when the fissure is large, renders the swallowing of fluids extremely difficult, the articulation painfully indistinct, and the sufferer peculiarly liable to the lodgement of foreign matter in the wind-pipe, and all the dangerous consequences arising therefrom.

To remedy these evils, an operation, denominated staphyloraphy, has been instituted, the credit of which there is reason to believe, belongs to M. La Monnier, a French dentist, who performed the operation some time previous to the year 1764. But it was left for the celebrated M. Roux to call the attention of the medical profession to the subject, which he did in the year 1819, since which time the operation has been performed by many surgeons in both Europe and America.

The operation consists in effecting a union of the cleft edges of the velum; and, in all cases, where the cleft extends no farther, and the patient is young, a most satisfactory result may be anticipated. But where the osseous palate is likewise involved, more or less of an aperture will of course remain, which must be closed either through the medium of granulations, or by a gold obturator or artificial palate before much benefit can be derived.

The earlier in life the operation can be performed the better. However, it is not often attempted much before the age of maturity, because, it is thought essential to have the aid and entire consent of patients while operating, and also that they be subjected to great care and self-denial during the remainder of the cure. But experience shows, that adults have, in fact, no command over that troublesome and continual motion of the parts, which always is encountered at every step of the operation; and that their consent is nothing more than to suffer their mouths to be kept sufficiently open, and their heads moderately still. Now where is the child of nine or ten years of age that could not be prevailed upon to do as much, and where is the operator of experience that would expect of any patient much more. Besides this, the operation cannot be deemed painful in its nature, nor one that requires much care and self-denial during the progress of the cure, while its effects upon the articulation, when performed in childhood, are inestimable, but if upon adults, the improvement is sometimes scarcely perceptible.

In cases, where patients have arrived at the age of maturity, their habits of speech become so firmly fixed, that it is almost impossible for them ever to overcome that snuffling, uncouth, hollow tone, so peculiar to those affected with openings in the palate. They have, too, in some measure, outlived their affliction, and cease to be annoyed at the attention their infirmity attracts, or to feel that degree of mortification they so sensibly experienced in their youth. They are apt, therefore, to lack that determination and watchfulness so necessary to overcome a fixed habit of any description, and more particularly the one under consideration.

But if the operation be performed in childhood, a period when the articulation is always attended to and corrected, from time to time, in every child, a perfect natural power of speech may be certainly attained. The better development of the parts may do much; the process of education will effect more; but there is an innate desire in every youthful mind to appear in society, and to the world, free from malformation of every kind, which is a never failing stimulant, sufficiently powerful to accomplish more towards a correct enunciation, than all the rest. I, therefore, greatly prefer performing staphyloraphy on children of nine or ten years of age. I have operated with success on a little girl only nine years old, and with less difficulty than I have frequently experienced on adults.

As a general rule, it is not necessary to diet or reduce patients before the operation. But it is always very important that the velum should be handled, from time to time, until the irritability of the parts are, in a great measure, overcome, before the operation is attempted.

The apparatus necessary for the operation, consists of a pair of slender curved forceps, six or seven inches long, the beaks coming together only at the point, and terminating in fine sharp teeth.

Also, a spear-shaped knife; the blade one inch long, and nearly one inch wide, being very thin and sharp, guarded at the heel, and mounted on a handle five or six inches long; six needles, each half an inch long, with a shoulder near the point, and tapered from the shoulder to the eye, the points having a triangular or three-edged form. Also, a needle-holder, on the principle of Dr. Hosack's *porte-aiguille*, but greatly simplified and improved.

The needle-holder is composed of two parts, a staff and a slider. The staff is round, six inches long, with an arm at the top, half an inch long, standing at right angles from the staff. Near the end of the arm is a hole in which the needles are fixed, and from the end of the arm to the hole is a narrow slit, through which the ligatures are passed in and out as occasion may require. Two inches from the lower end of the staff, a pair of rings are affixed to receive the thumb and middle finger, the rings standing parallel with the staff, and sideways to the direction of the arm. The slider is cylindrical in its form, of the same length as the staff, and is made to fit over, and to be moved up and down upon it. The upper end of the slider is split for an inch or more like that of a pen. One half inch of this split end stands off at a right angle, exactly under the arm of the staff, and forms a *clasp* to receive and extract the needle from the arm. A hole opposite the one in the arm of the staff is situated between the jaws of the clasp. Through this hole the point of the needle enters, opens the clasp as it protrudes, until the clasp closes behind the shoulder on the needle. The uniform direction of the clasp is governed by the rings on the staff, they acting as guides in slits made for this purpose in the slider. Also, an instrument for depressing the tongue, a pair of common curved scissors, three strong silk ligatures, each one yard long, double and well waxed, a hard piece of cork to place between the patient's jaws, three or four swabs, or pieces of sponge, mounted on a small-rod of whalebone, a basin of cold water, napkins, and a cloth to cover the patient.

The patient being placed on a low seat, in a good light, and in a reclining position, the breast covered with a cloth, the mouth open to its full extent, and a cork well adjusted between the jaws at the last molar teeth ; the operator, kneeling in front, may commenee the operation by seizing the left edge of the cleft, at the base of the half uvula, with the forceps, which is to be held firmly and steadily in the left hand. Then holding the spear-shaped knife, like a pen, in the right hand, the point may be introduced into the velum half an inch back from the palate-bone, and the sixteenth of an inch from the cleft-edge, and then plunged through to the guard backwards and towards the pharynx. Thus, in an instant, the edge is severed in a straight narrow strip forwards to the palate bone, and backwards, near to the uvula. The next moment the back part of the velum is to be drawn slightly forwards, and with one clip of the curved scissors the remaining portion of the edge may be cut away. The cork is now to be removed from between the jaws, and the patient is allowed to rest until the bleeding in a great measure subsides. The coagulated blood and mucus is then washed away from the velum and pharynx. The cork is then replaced between the jaws, and the other edge of the cleft is removed in the same manner as the first. It now only remains to cut the detached strips loose from the palate-bone, and the paring of the edges will be finished. After the bleeding has again subsided, and the blood and mucus is again cleansed away, the next step is to insert the ligatures.

The ligatures being double, with a staphyloraphy needle on each end, and having been put single through the eye of the needle, and the ends tied, the knot placed near one of the needles, so that it can be pulled through the velum, when the stitch is made, may be inserted very expeditiously, and at the very place desired, in the following manner: one of the needles with its ligature is fixed in the arm of the needle-holder, the point looking downwards, and the clasp, that is affixed to the slider, placed half an inch back from the point of the needle. Then replacing the cork between the jaws of the patient, and introducing the needle-holder deep into the mouth, passing the needle behind the velum and the clasp in front of it, the operator, fixing his eye on the hole in the clasp, and placing the hole about a quarter of an inch back from the raw-edge, and at the base of the uvula, pushes the slider suddenly upwards, carrying the clasp forcibly against the velum, through which the needle passes, enters the hole in the clasp, is there retained, and at the same instant drawn out from the mouth with one of the ligatures. The needle on the other end of the ligature is now fixed in the arm of the staff, and passed through the edge on the other side of the cleft at the same point, and in the same manner as the first. A second ligature is then inserted, in like manner, a quarter of an inch from the raw-edge, and about the middle of the velum, and, also, a third one in the same manner, one-fourth of an inch back from the palate-bone. As each ligature is inserted, the ends may be put out of the way of the operator by turning them behind the ears of the patient. The next step is to bring the edges of the velum together, and tie the ligatures.

The coagulated blood and mucus being well cleansed from about the ligatures, the velum and the pharynx, and the cork again replaced between the jaws of the patient, the operator, selecting the ends of the posterior ligature, puts the surgeon's knot upon it, and then carries the knot down before the ends of his forefingers to the velum, draws its raw edges neatly together, and there holds them, until an assistant puts a second knot upon the ligature, conveys it down to the operator's fingers, who, at at the same instant seizes it and carries it down securely upon the first knot. The second and third ligatures being tied in like manner, it only remains to cut off the ligatures about one-fourth of an inch from the knots, and the operation will be finished.

When, however, the cleft extends to a greater or less degree through the palatine arch, it frequently happens that the edges of the velum are carried so far apart, that it is impossible to bring them together without causing too great a tension upon the second and third ligatures. In such cases a transverse incision may be made along the posterior edge of the palate-bone on both sides of the cleft, and through the entire thickness of the velum, and to such an extent as to permit the raw edges to be properly approximated. In accomplishing this, should it even be necessary to make the incisions five or six lines deep, they will always heal up spontaneously, without causing the slightest apprehension or difficulty.

The ligatures being tied, nothing more remains to be done during the process of the union of the edges, but to guard against every thing that would probably induce the patient to have a "spell" of vomiting, coughing or sneezing. The consequences of talking and of deglutition, so much dreaded and forbidden, in the strictest manner, by most operators, I do not esteem to be the least injurious in any stage of the cure. In the summer of 1837, I performed the operation on a Mr. G. of Pennsylvania, aged about nineteen, and was under the impression that I was keeping him under the restrictions of "no talking, no eating," and but little drinking; when upon entering his room at an unusual hour, and on the very day that I had removed two of the ligatures, I found him regaling himself upon a plentiful supply of crackers and cheese, which he had procured at a neighboring grocery. He afterwards confessed that he had indulged his appetite very often in the same way during the whole process of the cure, and, I must add, without the least injury to his palate. Since then I have always allowed my patients a full supply of proper food and drinks, and liberty to talk as much as they might wish, and I have yet to witness the first untoward event arising from either cause.

The proper time to remove the ligatures will depend in a great measure upon the amount of ulceration that may exist, at the time, about them. As a general rule, the middle ligature may be removed on the third day, the upper upon the fourth or fifth, and the lower on the fifth or sixth day after the operation. This done, no further treatment will be required.

After the velum has been successfully united, if an opening still remains in the palatine arch from the cleft extending into it; and if the aperture is narrow, and its edges free from any upward inclination, it may be closed through the medium of granulations, without any reference to the length. But where the cleft extends through the alveolar arch, or where the edges of the cleft are turned upwards, even in a small degree, there is no other mode of closing the opening properly but with a gold obturator or artificial palate.

The process of closing an opening by granulations in the palatine arch, consists in rendering the edges of the opening raw, without causing, if possible, any loss of substance, and, at the same time, inducing such an amount of inflammation in the parts as will insure a large and speedy supply of granulations. The knife then, of course, cannot be resorted to with much hope of success. Caustics are more suitable, yet will not meet all the indications desired, without great care and trouble. But the actual cautery, so shaped as to permit its application to every part of the edge, and heated just sufficiently to blister freely, but not to sear, fulfils every indication required. The form of the cautery that will be required for this purpose, is simply a small iron instrument, not longer than a common probe, with a bulb on one end half an inch long and about a quarter of an inch thick. A deep groove or hollow is filed out around the middle of the bulb, so as to allow it to come in equal contact on every part of the edge to which it may be applied. The cautery is bent at a right angle near the bulb, and the other end well secured in a handle. The bulb of the cautery may be heated until it is slightly red, then, at the same instant, depressing the tongue with the index finger of the left-hand, it may be applied by first drawing it along the edge of the opening on one side, and then on the other, until the desired effect is produced; after which, no further treatment is usually necessary until six weeks or two months have elapsed, and then the cautery may again be resorted to, and so on until the opening becomes entirely closed. As the opening decreases, the size of the cautery must, of course, be reduced. In this manner I have succeeded in closing several openings, of greater or less extent, in the palate, and, in one case, where the cleft extended through the entire palatine arch.

The manner of constructing gold obturators or artificial palates, is so generally known, that I do not deem a description of them at this time necessary. They are often required from several causes, and when properly formed and adapted to the parts, may be worn with great comfort and benefit to the patient.

## Figures and Tables

**Figure f1:**
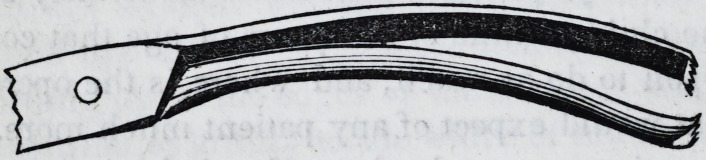


**Figure f2:**
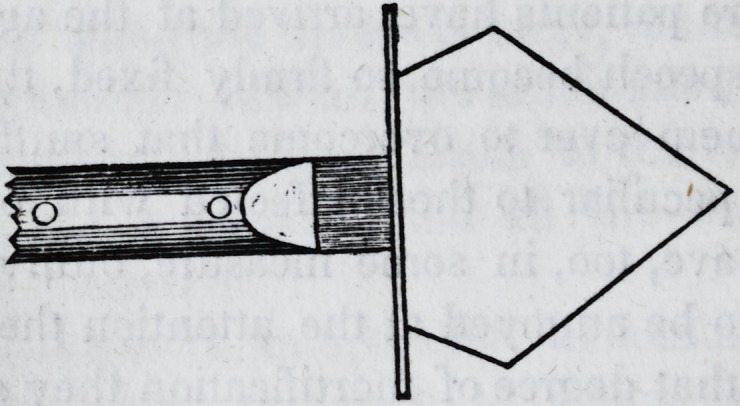


**Figure f3:**
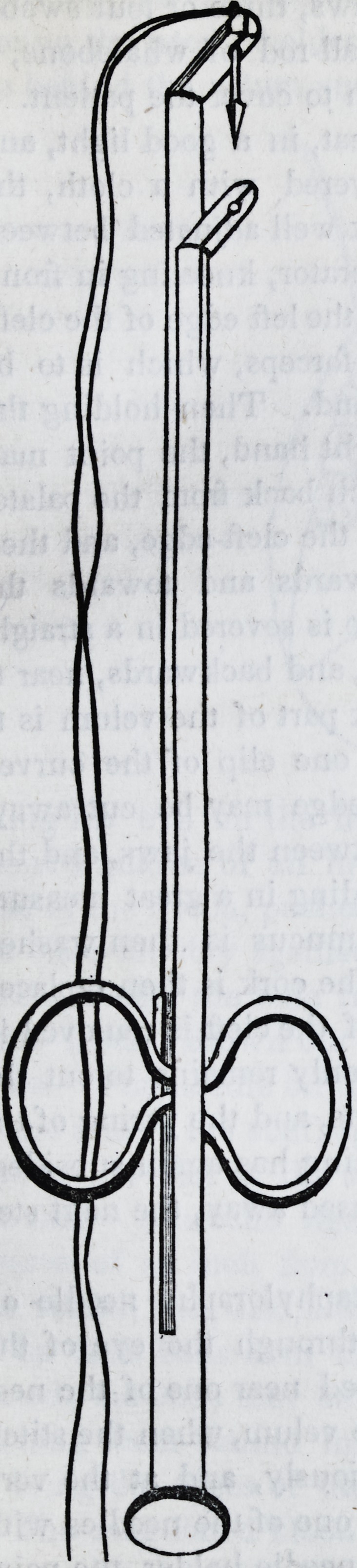


**Figure f4:**
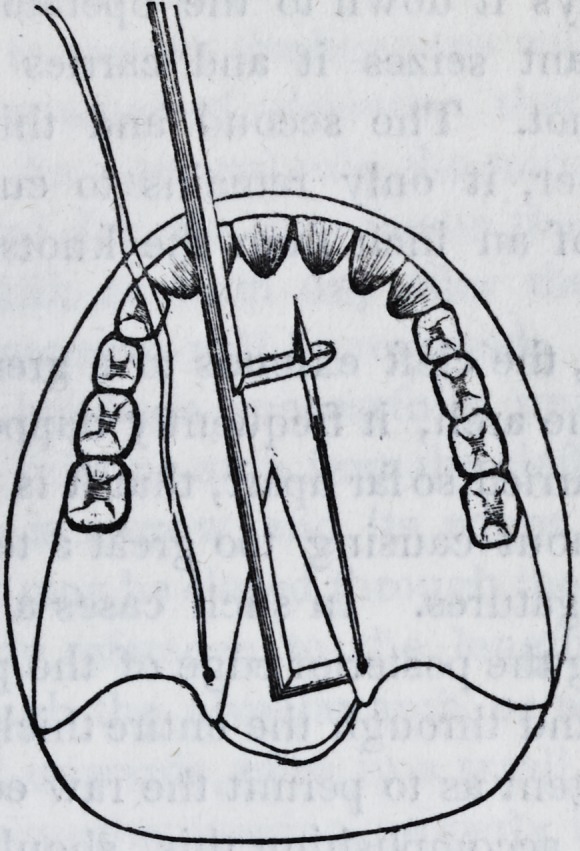


**Figure f5:**